# Social capital and community resilience in disaster contexts: a multiple case study from Colombia

**DOI:** 10.3389/fpubh.2026.1830318

**Published:** 2026-05-26

**Authors:** Luisa Fernanda Duque Monsalve, Paula Andrea Valencia Londoño, Carlos Arturo Sandoval Casilimas, Natalia Posada-Pérez, Milton Andrés Rojas-Betancur, Gloria Esperanza Londoño Torres

**Affiliations:** 1 Faculty of Psychology, University of San Buenaventura Medellín, Medellín, Colombia; 2 Institute of Social Science and Humans, Universidad de Medellín, Medellín, Colombia; 3 Colegio Mayor de Antioquia, Medellín, Colombia; 4 Institute of Social Science and Humans, Universidad de Medellín, Medellín, Colombia; 5 Colegio Mayor de Antioquia, Medellín, Colombia

**Keywords:** flash flood, social capital, community resilience, disaster risk management, multiple case study

## Abstract

**Introduction:**

This study analyses the role of social capital in community resilience to disasters. Although there is broad consensus on its importance, there is still a need to distinguish the specific contribution of its different forms (bonding, bridging, and linking) throughout the phases of the risk management cycle (preparedness, response, recovery, and mitigation).

**Methods:**

To address this gap, a multiple case study with a qualitative approach was developed, comparing three communities exposed to hydrometeorological risk in Colombia: Salgar and Mocoa, municipalities impacted by the country’s deadliest flash floods in the last decade, and La Primavera, a community with high exposure to risk where a major disaster has not yet occurred.

**Results:**

The findings confirm the centrality of social capital in community resilience throughout all phases of the risk cycle and broaden understanding of the phenomenon by identifying psychosocial processes, such as collective memory and social learning, that mediate this relationship. The analysis shows differentiated and complementary effects of each type of social capital evidencing that the sustainability of resilience depends on its dynamic articulation within collaborative risk governance frameworks. Likewise, the results suggest that, although disasters can weaken binding social capital, the bonds that persist constitute the basis for rebuilding the social fabric through solidarity practices and shared norms.

**Discussion:**

The study shows that social capital is an important condition for community resilience, but not sufficient on its own, as resilience also depends on other types of capital and on institutional and governance factors. In this regard, limits and ambivalent effects of social capital on risk management are identified. Finally, the research contributes to the literature by highlighting its relevance in the mitigation phase, which has traditionally been less explored.

## 1 Introduction

Disasters are critical events that transcend the immediate destruction of human lives, infrastructure, and livelihoods. They transform social relations, community ties, and forms of territorial governance, altering the networks that sustain collective life. These adverse effects manifest themselves over the long term in contexts characterized by structural inequalities, institutional fragility, and high exposure to risk—contextual conditions that are common in Latin America.

From a disaster risk management perspective, community resilience has become established as a fundamental analytical category for explaining why some communities manage to reorganize, adapt, and recover more effectively than others in the face of recurring adverse events.

Various studies agree that resilience is a continuous process throughout the entire risk cycle, including the mitigation, preparedness, response, and recovery phases, and define it as a community’s ability to anticipate, absorb impacts, adapt to changing conditions, and transform its social, institutional, and territorial structures in the face of real or potential threats; becoming what enables communities to move from improvised responses to sustained processes of social learning and vulnerability reduction (1).

Some findings indicate that, in addition to infrastructure, technical resources, and institutional capacities, social relationships are a key component of community resilience. Support networks, cooperation, trust, and collective organization facilitate coordination in crises, reduce unequal exposure to risk, and enable the mobilization of resources when the state response is limited. These same results indicate that social capital is a fundamental factor in explaining resilience to disasters, as it promotes mutual aid, information flow, shared interpretation of threats, collective action, and coordination between community and institutional actors, especially in communities with strong cultural identities and high levels of cohesion (2–8).

Along the same lines, recent Latin American studies relate to the above, specifically regarding the link between public policies, social capital, and resilience (9–11); and the interdependence between social capital and urban policies, which is reflected in community resilience insofar as it depends, among other factors, on community-state articulation (12).

Similarly, Latin American studies on risk management emphasize relational and communicative processes as the foundation for community resilience (13), which in turn shape social capital understood as collective organizational capacity (14, 15) and an expression of the collectivization of resources to efficiently address crises. These trends are replicated in Colombia, where cases have been documented in which communities rely on social and community networks to cope with disastrous events, given their distrust of institutions (16). Furthermore, the value of institutional social capital in the solidarity economy sector and its relationship to community resilience are highlighted, such that the trust and cooperation that emerge in these spaces improve the capacity to respond to risk (17)). Although there is no broad consensus on the definition or the best strategies for measuring it, the literature recognizes the existence of different types of social capital. On the one hand, there is cognitive social capital, associated with shared perceptions such as trust, norms of reciprocity, and a sense of belonging; on the other hand, there is structural social capital, linked to network density, community participation, and collective organization; and relational social capital, referring to the quality and stability of social ties. Complementarily, according to their social function, bonding social capital, bridging social capital, and linking social capital are identified, categories that allow for the analysis of intra-community, inter-community, and institutional relationships, respectively (3, 4).

Studies also indicate that the performance of social capital depends on the institutional context and forms of governance that mediate the relationship between communities and the state. In contexts where these connections are weak, community social capital can partially compensate for institutional deficits, but it can also become overburdened and eroded by state inaction (18–20).

There is also evidence, produced by longitudinal studies, indicating that disasters can erode social capital, especially cognitive capital, which introduces a critical dimension into the analysis of community resilience and challenges overly normative views of social capital as an always positive and ever-present resource (3, 21, 22).

However, although social capital is widely recognized as a central element of community resilience, analytical gaps remain. Most studies focus on the global north, leaving contexts with institutional fragility less studied. Previous studies have concentrated on post-disaster scenarios (23) and there is little evidence on its role in risk mitigation, compared to other phases of disaster (4). Likewise, a predominance of structural indicators of social capital (e.g., networks) has been noted, to the detriment of its psychosocial (e.g., social identity) or cognitive dimensions (23, 24). Furthermore, the differentiated contributions of the types of social capital and their possible negative effects, such as internal tensions or community conflicts, have not yet been explored in depth.

Based on these considerations, this study revisits the premise of Aldrich and Meyer (25), according to which community resilience to disasters does not depend exclusively on investment in physical infrastructure, but rather on the following fundamental factors: the strength of human relationships, support networks, and reciprocity mechanisms.

**Figure 1:**
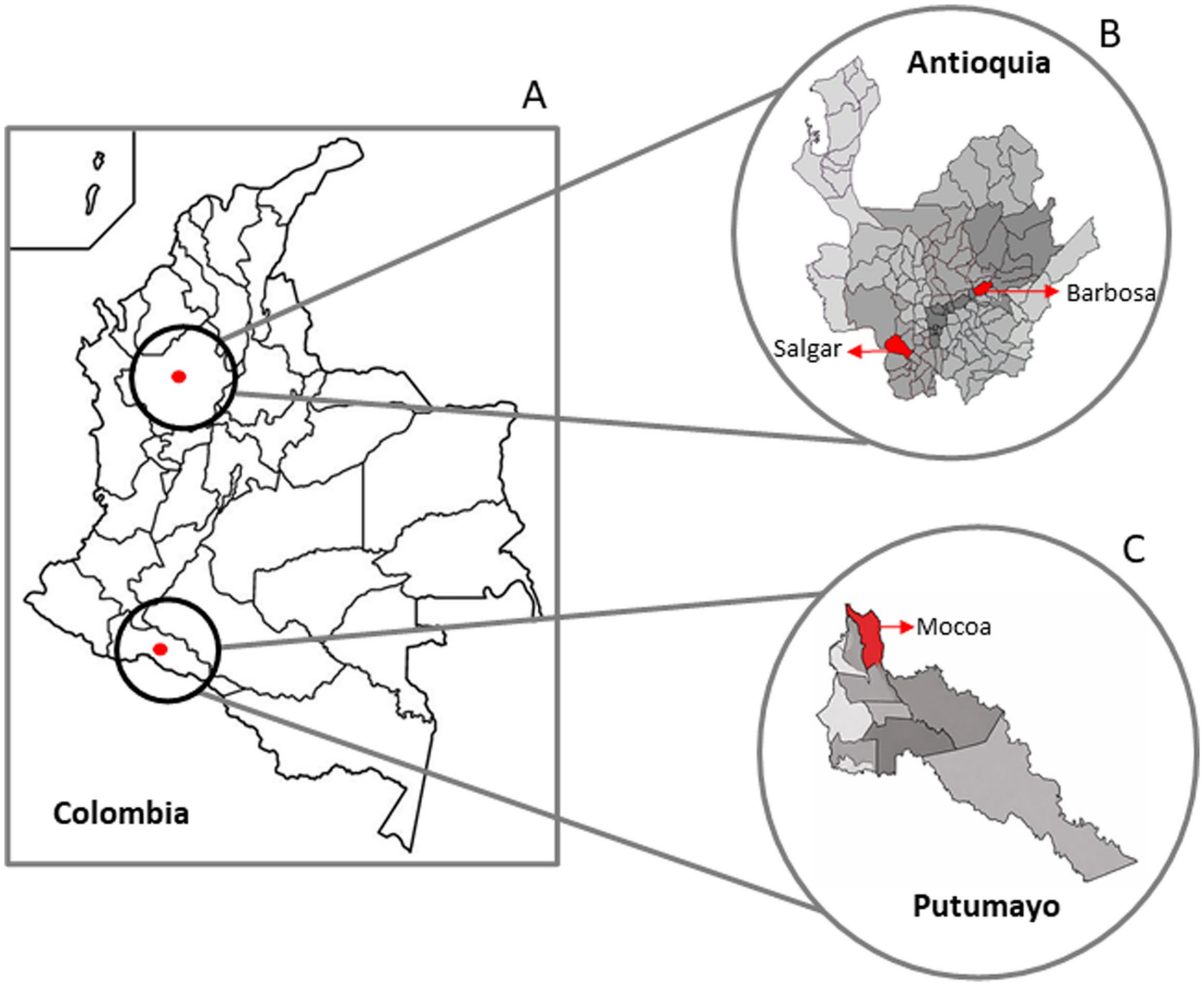
**(A)** Location of the study areas in Colombia. **(B)** Location of the municipalities of Barbosa and Salgar in the department of Antioquia. **(C)** Location of the municipality of Mocoa in the department of Putumayo.

Within this framework, this study analyzes how social capital promotes or limits community resilience to disasters at different stages of risk management, based on a multiple case study in three Colombian communities: Salgar (Antioquia), Mocoa (Putumayo), and the village of La Primavera (Barbosa, Antioquia). These cases were selected for their exposure to hydrometeorological threats, the occurrence of disasters of varying magnitudes, and the heterogeneity of social and institutional contexts, which allowed for a comparative analysis of the dynamics of social capital in urban, semi-urban, and rural settings (Figure 1).

The main contribution of this article is that it offers an integrated view of social capital throughout the entire disaster risk management cycle, explicitly incorporating the mitigation phase, which is traditionally underrepresented in the literature (4). The study also provides qualitative empirical evidence to understand how different forms of social capital interact with each other and with the institutional context, showing both their potential and their limitations. In particular, dynamics are identified in which binding social capital compensates for state deficiencies, bridging social capital expands support networks beyond the territory, and linking social capital is decisive, although not always effective, in guaranteeing rights and implementing public policies for disaster risk management.

Finally, the relevance of this research transcends the academic sphere. In a country such as Colombia, where disaster risk management faces persistent challenges in territories marked by structural vulnerabilities, the findings offer empirical inputs for the design of public policies that recognize and strengthen existing community capacities. Understanding the role of social capital not only improves the effectiveness of institutional interventions, but also advances toward more inclusive, participatory, and territorially sensitive approaches to resilience, capable of articulating infrastructure, governance, and social fabric in the sustainable reduction of disaster risk.

## 2 Methods

The research project falls within the interpretive paradigm, insofar as it aims to understand the meanings and senses of human action as historical and contextual constructions (26). In line with this epistemological position, the study adopted a qualitative approach and used the case study method, which is particularly suitable for analyzing complex phenomena closely related to their context, such as the contribution of social capital to community resilience.

A multiple case study design was adopted, as this allows for cross-analysis and the construction of more robust and nuanced understandings of the phenomena of interest (27). The temporal delimitation of the cases covers community resilience processes before, during, and/or after disasters, which made it possible to examine comparatively how different types of social capital contribute at different moments in the risk management cycle (27). In Stake’s terms (28), this is a collective case study, in that the research interest focuses on a general phenomenon, the relationship between social capital and community resilience, and, in order to understand it, several cases are selected and analyzed in depth. In this work, the case is not defined as the disaster event itself, but rather as a situated social process in which social capital and community practices related to disaster risk management are articulated. Each case was defined as a geographically localized community exposed to hydrometeorological threats, in which social capital was analyzed in relation to community resilience to disasters, both in contexts of latent risk (ex ante) and in post-disaster scenarios (ex post).

In order to maximize the analytical potential of the comparison, cases were selected using purposive and theoretical sampling. Consistent with the replication logic proposed by Yin (27), the selection combined criteria of similarity in key conditions (literal replication) and theoretically relevant contrast criteria (theoretical replication).

In order to make rigorous comparisons, it is necessary to keep the type of phenomenon analyzed constant (literal replication). For this reason, cases exposed to risks of the same nature were selected, which is consistent with the findings of previous studies that indicate that community resilience processes vary according to the type of disaster (29). This study focused on hydrometeorological disasters, given that they are the most frequent and severe in Colombia, taking into account the human, social, and material impacts they generate (30).

Following the logic of theoretical replication, cases were also selected for their differences in key components, especially in terms of exposure to the disaster, with a view to generating interpretatively meaningful contrasts. Given that scientific literature recognizes that community resilience is not limited to the post-disaster phase, but also includes anticipatory dimensions such as disaster prevention, in what some have termed proactive resilience (24), the design incorporated both post-disaster scenarios (Mocoa and Salgar) and a context of latent risk without recent disaster occurrence (La Primavera). This combination made it possible to examine how different forms of social capital are configured and mobilized at different stages of the risk management cycle, broadening our comparative understanding of the phenomenon. Descriptions of the three cases are provided below.

La Primavera is a rural settlement in the municipality of Barbosa, comprising approximately 300 families, most of whom are victims of forced displacement. Its location, between the Medellín River and the Northern Freeway, configures a scenario of high territorial vulnerability. The community faces recurring risks of flooding and fires, as well as threats of eviction associated with infrastructure projects, particularly the construction of the commuter rail line. This context represents a case of latent risk (ex ante), in which community resilience is articulated primarily through a preventive and preventive and organizational framework.

The municipality of Salgar, located in southwestern Antioquia, was the scene of a torrential flood on May 18, 2015, which caused 104 deaths and serious social and material damage. Currently, nearly 80% of its territory is at high risk of windstorms, fires, torrential rains, landslides, and floods. It is a coffee-growing municipality affected by the internal armed conflict that has been ongoing in Colombia for more than five decades.

Mocoa, capital of the department of Putumayo, was devastated on March 31, 2017, by a torrential flood that left 335 people dead, hundreds injured and missing, as well as thousands homeless and severe damage to infrastructure. The municipality has also historically received victims of forced displacement and is characterized by marked cultural diversity, with a significant proportion of its inhabitants belonging to indigenous communities.

In qualitative case studies, participants’ narratives are a fundamental means of understanding the experiences, meanings, and perspectives of social actors (31). Consistent with this approach, information was gathered through 96 semi-structured interviews, 10 focus groups, as well as with participant observation records developed in each of the contexts studied. Table 1 presents the details of the types of participating actors in each case, according to the data generation technique and their respective numbers.

**Table 1:** Data collection techniques across cases.

Techniques	Primavera	No.	Mocoa	No.	Salgar	No.
Semi-structured interviews	Social leaders: community action board and asociación de víctimas los meandros nuevo amanecer	9	Social leaders: Community Action Boards, citizen oversight committees, environmental collectives	15	Social leaders: Community Action Board, environmental, cultural, and victims-of-armed-conflict collectives	6
	Community members residing in the “upper,” “middle,” and “lower” areas of the settlement	21	Community members: residents from different neighborhoods and Indigenous councils (cabildos) of Mocoa, as well as from the Sauces I housing development (resettlement project)	5	Community members: residents of “La Habana,” “La Florida,” “Las Margaritas,” and “La Aldea” (housing–resettlement projects)	21
	External actors: public officials, emergency response agencies, representatives of companies operating in the territory, NGO staff, and university personnel with presence in the territory	9	External actors: public officials, business actors/subcontractors involved in reconstruction projects	4	External actors: public officials, emergency response agencies, NGOs with presence in the territory	6
Focus groups	Social leaders and community members participated in participatory mapping exercises (“talking maps”) to identify threats, construct timelines of resilience and family/community vulnerability to disasters, map key stakeholders for risk management, and analyze leadership and community organization processes	7	Not conducted (territorial distance prevented sustained field presence and participant recruitment)	N/A	Social leaders and community members participated in participatory mapping exercises (“talking maps”) to identify threats, construct timelines of resilience and family/community vulnerability to disasters, and map key stakeholders for risk management	3
Participant observation	Field visits guided by social leaders; health promotion activities; farmers’ markets; meetings of the inter-institutional committee (with active participation of researchers); community meetings with state entities responsible for disaster risk management (DRM)	N/A	Field visits guided by social leaders; dissemination of the aqueduct reconstruction project; political campaign events of social leaders running for the municipal council; meetings of citizen oversight committees	N/A	Field visits guided by local authorities	N/A

Source: authors’ own elaboration.

All participants provided their written informed consent, and the study was approved by the Ethics Committee of the University of San Buenaventura, ensuring compliance with the ethical principles of research involving human subjects^1^. Sample sufficiency was determined based on three criteria: 1. Literal and theoretical replication logic: The selection of cases and participants was guided by the aim of assessing whether findings were consistent across cases (literal replication) or varied according to the anticipated theoretical conditions (theoretical replication) in relation to the research questions. 2. Triangulation and convergence of evidence: Multiple data generation techniques and diverse actors were incorporated. In La Primavera and Salgar, focus groups functioned as a mechanism to corroborate information previously obtained through interviews (conducted in an initial phase of fieldwork). In Mocoa case, it was not possible to conduct focus groups (geographical distance limited prolonged field presence, which hindered the organization of such activities). 3. Representation of key roles and actors: Particularly in the communities of Salgar and La Primavera, extensive coverage was achieved by interviewing all identified social leaders and key actors in disaster risk management who agreed to participate voluntarily. For community members, representation was ensured across geographical sectors (upper, middle, and lower areas in La Margarita) and across the four resettlement projects in Salgar. In Mocoa, although access was more complex due to the size of the population and logistical constraints, the sample was composed of those actors who agreed to participate voluntarily, ensuring that the critical profiles required to address the research questions were represented.

After transcribing the audio recordings of interviews and focus groups in full, the data were processed using coding and categorization procedures typical of qualitative research, aimed at identifying patterns and concepts relevant to answering the research question. Following (32), two cycles of coding were conducted. In the first cycle, three researchers independently coded the data after dividing the material among themselves. This phase followed a provisional (deductive) coding strategy, based on a predefined codebook developed through a review of the literature and agreed upon by the research team. The codes applied in this stage included descriptive, process, structural, emotion, and values coding (32). Throughout this process, the researchers maintained ongoing communication to incorporate emergent codes, refine the codebook, and reach consensus on coding decisions in response to questions and ambiguities that arose during analysis. Subsequently, the lead author of this article—who had participated in the initial coding—conducted a second cycle of coding. In this phase, codes were refined through cross-case comparison and a more in-depth engagement with the scientific literature. This stage employed pattern, axial, and focused coding strategies (32), resulting in the development of higher-level categories and subcategories. During the second cycle, codes were continuously discussed and revised through consensus with another member of the research team who had not participated in the first coding cycle. All coding procedures were supported by the use of Atlas.ti (version 25). The resulting category map is presented in Supplementary Material S1.

According to Yin (27), coding and categorization allowed for the systematic organization of the evidence produced during fieldwork, facilitating the application of analytical strategies specific to case studies, such as pattern comparison and the construction of interpretive explanations (non-causal, given the qualitative nature of the study), time series analysis, the use of logical models, and the synthesis of cross-cases. These analytical strategies gave rise to the results presented in this paper.

To ensure the integrity of the analytical process, the study was conducted by a multidisciplinary team of scholars in psychology, political science, and territorial studies. This group brought substantial experience in community-engaged research and specialized expertise in the production and interpretation of data, particularly regarding social capital and community resilience. While maintaining an external researcher position, the team possessed substantive familiarity with the historical events, socio-political contexts, and institutional frameworks governing risk management in the three Colombian case studies: Salgar, La Primavera, and Mocoa. Relationships with these communities were cultivated through collaborative networks and participatory methodologies, while strategic engagement with institutional and social actors facilitated access to critical information and established the trust essential for fieldwork. The authors acknowledge, however, that disciplinary backgrounds and interactions with specific stakeholders may influence the interpretation of social capital dynamics and community-led governance. These considerations were addressed through systematic reflexive practice throughout the investigative process. By integrating this professional expertise with a rigorous self-aware approach, the methodology provides a robust foundation for the findings discussed in the following sections.

## 3 Results

The following describes how, in the three case studies, social capital has contributed to community resilience in different disaster risk management processes.

### 3.1 Social capital in emergency preparedness

#### 3.1.1 Knowledge of disaster risk

Disaster risk management requires communities to recognize and understand the hazards to which they are exposed, understanding that this perception is a collective process, built through shared interpretation of threats and vulnerabilities, and enhanced by community communication and social ties.

In all three cases, there is a clear perception of risk, resulting from collective learning and socially embedded knowledge. In Mocoa and Salgar, this perception intensified after the disasters, transforming the previous interpretation of rainfall, which had previously been considered harmless, and consolidating the recognition of the recurrence of disasters and the need to manage risk.

In Mocoa, the disaster triggered collective memory through the recovery of intergenerational stories that warned of torrential floods in areas that were later urbanized, turning that memory into a preventive tool: “The advice would be rather that people should not build near rivers, that they should respect the history of their grandparents...” (I_LL_M_01).

In La Primavera, the perception of risk emerged from direct observation of changes in the riverbed, particularly its proximity to homes, and was reinforced by social capital through the support of NGOs and companies: “*With Corporación Región, we highlighted these risks and raised awareness*.” (I_LL_P_01).

In all three cases, local knowledge about the territory is combined with technical expertise provided by state entities, which is reflected in the community’s appropriation of technical concepts and the construction of shared frameworks of interpretation. In La Primavera, specialized studies confirmed community perceptions of risk: “*The resettlement is necessary because people are living in an area at risk of flooding (...) the study by the National University justified it*.” (Interview with state actor La Primavera1). In Salgar, the dissemination of scientific knowledge helped to dispel initial religious interpretations of the disaster: “*that it was God’s punishment*” (I_LL_M_02).

The increased perception of risk has led to daily practices of observing and analyzing river behavior, demonstrating collective and situated learning processes: “*When the river rises, you feel calm (...) but when it rains and it doesn’t rise, you get scared because you think it’s damming up*.” (I_R_S_01).

#### 3.1.2 Disaster risk communication

Early warning systems (EWS) have been implemented in all three communities to monitor rivers. In La Primavera and Salgar, these systems remain operational thanks to municipal and departmental investments, while in Mocoa they are not currently in use. In Salgar and Mocoa, the EWS were installed after torrential floods, while in La Primavera they were set up on the initiative of the community, with support from NGOs and public entities.

The installation of the SATs was accompanied by training processes and the formulation of community risk management plans. However, their operation has limitations. In La Primavera, some residents report not hearing the alarms or being unaware of the procedures, and in Salgar, there has been evidence of poor performance in events that required their activation: “*There have been very heavy downpours, which should have triggered the alarms, but they never went off* (I_LL_M_02).

In Mocoa, although the system was activated during torrential flooding in 2018 and helped prevent further damage, it stopped working shortly afterwards: “*They invested 1.2 billion in an alarm system (...) that lasted (...) a year at most (...) the sirens (...) didn’t sound*.” (E_M_L_2). In Mocoa, these failures highlight problems of intergovernmental coordination. The municipality did not take over the operation of the SAT installed by the national authority, arguing that the system already required costly maintenance at the time of delivery. Added to this was the withdrawal of the UNGRD (National Disaster Risk Management Unit) from the territory after the change of government, which interrupted the flow of technical information to the community: “*It has been more than a year since the National Unit left (...) they sent us the curve lines that the system sent*.” (I_LL_M_02).

Given these institutional shortcomings, state training programs have sought to strengthen community autonomy in interpreting monitoring indicators, a visible appropriation in Salgar and La Primavera: “*We have to be alert because suddenly the alarms may not be working (...) so we go to the river to check*.” (I_R_S_02). In all three cases, the SATs incorporate “river watch” programs, which are responsible for monitoring and communicating risk.

During the tragedies in Salgar and Mocoa, the most effective warning strategy was solidarity among neighbors, through word of mouth and WhatsApp, supported by binding social capital: *“They started knocking on neighbors’ doors, alerting that small sector.*” (I_R_S_02). In La Primavera, where the community is smaller and more cohesive, community ties facilitate both institutional activation and neighborhood mobilization for evacuation and emotional support, with local leaders playing a central role: “*The alarm sounds (.) we immediately communicate (.) we have to say ‘calm down, nothing is going to happen*.’ (Interview with social leader La Primavera 2).

In all three territories, there is a sustained practice of community surveillance of rivers and streams, intensified by fears of a recurrence of the disaster. Community members monitor water levels, disseminate information, and report alerts to the authorities: “Anything that happens with the river (...) they report it.” (I_SA_P_01). These practices became established in Salgar and Mocoa after the disasters and in La Primavera due to increased risk perception and the occurrence of smaller-scale events.

In Mocoa and La Primavera, community leaders also highlight risk situations in spaces for citizen participation; however, their demands often translate into limited institutional responses, such as technical visits or evacuation recommendations, which citizens perceive as insufficient given the magnitude of the risk.

In summary, social capital is decisive for the effectiveness of SATs: given the technical and institutional limitations of the devices, community networks based on trust, solidarity, and communication constitute a more robust basis for risk alerting and communication.

#### 3.1.3 Preparing for emergency response

The three communities have made progress in building disaster response capacities, mainly through training processes promoted by state entities and cooperation organizations with a presence in the territory. These processes have resulted in evacuation drills, route signage, identification of safe areas and meeting points, first aid training, and the participatory formulation of Community Risk Management Plans. When the SAT alarms are activated, community evacuation responses are generated, even for families not previously affected by disasters. In all three territories, some families have incorporated preventive practices into their daily lives, such as preparing emergency backpacks, organizing personal documents, and identifying key contacts.

It should be noted that in La Primavera, these capacities were developed proactively, without the prior occurrence of a disaster, while in Mocoa and Salgar they were mainly consolidated in the period following the tragedies. This case therefore highlights the importance of risk perception, community organization, and the empowering support of non-governmental organizations.

Although drills have been a key educational tool, their discontinuity and limited participation in the three territories have affected the adoption of protocols, especially among new residents, highlighting the role of social cohesion in the sustainability of preparedness processes.

In summary, the three cases demonstrate a recent strengthening of community capacities for risk management, based on participation, collective learning, and cooperation with external actors. However, challenges remain related to unequal participation and the continuity of training processes. Social capital has been fundamental to these advances, while the case of La Primavera highlights the role of social leadership and community organization in consolidating effective disaster preparedness strategies.

### 3.2 Social capital in the emergency response

#### 3.2.1 Response to torrential floods in Salgar and Mocoa

In Salgar and Mocoa, during the days following the torrential floods, binding social capital—family, neighborhood, and friendship networks—played a central role in the initial response to the emergency. In Mocoa, aid was primarily directed toward the family unit, revealing patterns of solidarity characteristic of this type of capital, which privileges strong internal ties and tends to exclude “others”: “People went for their families, and that’s absolutely normal.” (I_LL_M_01).

In Salgar, although solidarity was also concentrated in the immediate surroundings, it was articulated as a continuation of previous practices of mutual support in a context of structural vulnerability, where historically there have been dense networks between close neighbors. In both municipalities, relatives took in affected family members and organized forms of support among neighbors and friends through the exchange of food, clothing, accommodation, and emotional support. In Mocoa, these expressions were more intense in neighborhoods with greater social cohesion and strong cultural identities, such as those inhabited by indigenous communities. In Salgar, spontaneous networks of solidarity-based resource sharing also emerged, based on social capital bridged by citizens from unaffected neighborhoods.

However, evidence shows that these expressions of solidarity intensified in the immediate aftermath of the disaster and tended to weaken in the medium term, taking the form of *“momentary solidarity”* (Interview, Salgar 1 relief team). Likewise, in both cases, there were episodes of people taking advantage of the chaos and criminal acts, which highlights the limits of binding social capital in crisis contexts.

In Mocoa, in addition to family and neighborhood action, community organizations and pre-existing leadership played a strategic role in cooperating with the State during the response. Noteworthy examples include the mingas (community work parties) to restore community aqueducts and the role of the presidents of the Community Action Boards (JAC) in conducting the census of victims, a particularly complex task due to the high number of tenants and displaced persons. These dynamics are part of an urban context marked by the massive influx of victims of the armed conflict, which has led to Mocoa being characterized as a “city of refuge” (33).

In both municipalities, social institutions such as churches and schools played an important role in identifying and caring for victims. Likewise, social capital, based on connections with family, friends, and businesses in other regions of the country, facilitated the arrival of humanitarian aid, a process amplified by national media coverage.

Given the magnitude of the disasters, local institutional capacities were overwhelmed, making the response largely dependent on social capital networks. In both cases, the UNGRD led emergency planning, and the deployment of armed forces and external relief agencies was viewed positively. However, perceptions of the state response differ significantly between the two municipalities. In Salgar, the institutional response was widely regarded as effective. The proximity to Medellín (the country’s second largest city) facilitated an “oversupply” of humanitarian aid and rapid inter-institutional coordination, which made it possible to carry out rescue operations, provide medical and psychological care, search for missing persons, and restore basic services. In contrast, in Mocoa, there were reports of shortcomings in rescue efforts, the handling of bodies, the census of victims, and the distribution of aid. These failures led to the organization of volunteer rescue teams, citizen protests, and parallel censuses, as well as allegations of corruption, patronage, and hoarding of aid, involving both state agents and some social leaders. In response to these irregularities, community practices of solidarity-based redistribution and alternative leadership emerged. Although irregular practices and political use of aid were also mentioned in Salgar, these did not lead to formal complaints or organized citizen responses. Consequently, while in Salgar the state response was perceived as efficient, in Mocoa some participants describe a double tragedy: “*the natural and the institutional*” (I_LL_M_10).

These differences can be explained by several factors. First, the magnitude of the disaster was significantly greater in Mocoa. Second, Salgar has stronger social capital ties because it belongs to the department of Antioquia, one of the country’s main centers of power, while Putumayo occupies a peripheral position. Finally, in Mocoa, particularly strong social capital in terms of leadership and community organization partially compensated for institutional deficiencies, although it also led to a more critical citizenry and, consequently, lower satisfaction with the government’s response.

In summary, in both municipalities, binding social capital was key to the immediate response, although initially oriented toward the closest ties. In the stabilization phase, bridging social capital expanded support networks beyond the territory, while linking social capital showed substantive differences between the cases. Salgar illustrates the potential of an effective state response supported by strong inter-institutional links, while Mocoa highlights both the limits of state action in peripheral contexts and the capacity of community social capital to compensate for, denounce, and resist institutional failures.

#### 3.2.2 Response to new emergencies

Following the torrential floods, both Mocoa and Salgar have faced events of lesser magnitude, while La Primavera has experienced recurring floods that have caused partial or total damage to homes. In these cases, the institutional response follows a standard protocol that includes community reporting, initial response by emergency services, technical assessment, and a census of those affected, the delivery of humanitarian aid, the issuance of evacuation orders, and, in some cases, mitigation recommendations. These situations are managed by the Municipal Risk Management Council, which defines action plans and, when local capacities are insufficient, requests departmental or national support.

However, all three municipalities face institutional limitations associated with a shortage of personnel and resources, as well as the operational capacity of emergency services. In Salgar, some residents recognize the dedication of firefighters, while others question their preparedness for larger-scale emergencies: *You have to collect a dead body and debris, and then you say*, *“What about something bigger? How are they going to do that?”* (Interview, resident of Salgar 3). Despite the magnitude of previous disasters, neither Salgar nor Mocoa have a specialized risk management agency or fire departments equipped with capabilities commensurate with the levels of threat: “Here, I do see the need for a risk management department, or for budget to be invested in this, because the municipality needs it. Sometimes I can’t cope.” (I_SA_S_01).

There are also reports of delays in the delivery of humanitarian aid, which reinforces the perception of institutional inefficiency. In Salgar, the relief agencies themselves acknowledge these failures: “We get a lot of windstorms here (...) but we have to wait for the risk management people to get together, 15 days, 20 days, 30 days (...) it’s a failure” (I_ER_S_01). Furthermore, the aid is perceived as insufficient to guarantee decent living conditions, especially the rent subsidy, which is limited to three months and does not provide a clear path to permanent housing solutions. This situation generates resistance to evacuation orders and encourages people to remain in high-risk areas, as evidenced in La Primavera: “They come here with a piece of paper and say, ‘You have to evacuate’ (...) That she should find a house and that they would pay for it for three months. What is she going to do after three months?” (I_R_P_01).

In summary, although Salgar and Mocoa have partially strengthened their response mechanisms following torrential floods, structural limitations persist related to a lack of personnel, resources, and long-term planning, as well as the absence of sustainable housing solutions. The case of La Primavera confirms that, in the face of recurring events of lesser magnitude, the institutional response continues to be predominantly reactive.

### 3.3 Social capital in disaster recovery

#### 3.3.1 Recovery of physical and mental health

In Salgar and Mocoa, torrential flooding caused severe physical and mental health problems, especially among those who lost family members. In Salgar, the emotional impact was particularly profound because the most affected areas were made up of highly cohesive kinship and neighborhood networks, which led to multiple bereavements: “*We lost 11 family members*.” ((FG_R_S_03). The simultaneous loss of family members, friends, and acquaintances intensified the collective suffering in a small community where “everyone knows each other” (I_R_S_03). In this way, the pre-existing bonding social capital operated in an ambivalent manner: it was a source of solidarity and mutual support, but it also amplified the pain by concentrating losses within dense networks.

Symptoms of anxiety, depression, and post-traumatic stress persist in both municipalities, most notably in Salgar, where hypervigilance, re-experiencing of fear, and intense responses to stimuli associated with the event are observed. These manifestations are often accompanied by avoidant coping styles (emotional restraint). In Mocoa, psychological distress is also associated with unresolved material losses, which prolongs the feeling of insecurity and hinders the reconstruction of the Project of life.

Despite this, social capital also facilitated emotional recovery processes in both cases, through solidarity and daily emotional support among family members, neighbors, and friends: “It wasn’t just him and me, but the community.” (I_R_S_03). Churches and schools also served as spaces for emotional support: “Several churches joined together to provide psychological assistance.” (I_R_M_03). In La Primavera, these dynamics are expressed in sensitivity to the suffering associated with illness, old age, abandonment, or the effects of floods that have destroyed the homes of some families.

For its part, social capital links enabled access to psychosocial support from state entities, NGOs, and foundations. In Salgar, a relatively intensive and sustained strategy was implemented, with family, group, and individual support, which was well received by the victims. In Mocoa, although psychological first aid and group psychosocial care were offered, many participants perceived low coverage, short duration, and a bureaucratic orientation focused on compliance with indicators.

In both municipalities, the sustainability of the interventions was questioned. In Salgar, some residents pointed out that the repetition of stories about the disaster and the commemorations revived their pain. In this place, silence and avoidance emerge as culturally ingrained coping strategies, also associated with previous experiences of sociopolitical violence in southwestern Antioquia, where a “society of silence” characterized by fear, mistrust, and social withdrawal (34).

In summary, in Salgar, the psychological impact is profound and persistent, with unresolved grief and symptoms of post-traumatic stress, especially among those who lost family members and now find themselves in a state of resignation rather than well-being. In Mocoa, although progress has been made in emotional recovery, there are still cases of prolonged impact, highlighting the need for comprehensive and sustained interventions, coordinated with improvements in material living conditions. In all three cases, partial recovery of mental health has been supported by bonding, bridging, and linking social capital. However, institutional psychosocial support was perceived as insufficient, and in Mocoa as bureaucratic. Finally, the case of Salgar shows that the destruction of a highly cohesive social fabric can aggravate the psychological impacts of the disaster, especially when previously established avoidance coping styles predominate.

#### 3.3.2 Recovery of the social fabric

In Salgar and Mocoa, the disaster went beyond material losses and profoundly affected the social fabric, causing pain due to the disappearance of neighborhoods, community councils, and significant landscapes: “That whole area used to be homes (...) now it’s a place for drug dealing and consumption” (I_LL_M_06). Social fragmentation can be explained by the separation of families from their former neighbors and relatives, the loss of networks of trust, and the displacement of the population due to fear of new events: “*People left because they were physically afraid (...) many did not return”* (I_LL_M_09).

Following the resettlements, ties in both municipalities tended to be restricted to close relationships, weakening broader cohesion. In Salgar, some express community identification with the resettlement projects (new homes), while others express detachment and nostalgia for their previous neighborhood life: “*We miss that closeness (...) you no longer know whether to greet your neighbor or not”* (I_LL_S_04). In both cases, the loss of meeting places reduced everyday social interaction and accentuated loneliness: “The neighbourhood culture was lost” (I_EA_M_01); “We no longer have the same company (...) now it’s just us” (I_R_M_04).

Cultural and recreational life was also affected. In Salgar, community theater, dance, and music groups collapsed after the tragedy, and traditional festivals lost their central role: “Then it was like starting over, and they couldn’t sustain themselves” (I_LL_S_04). In contrast, in Mocoa, some institutions contributed to the rebuilding of the social fabric. Schools offered support to students and families, and community action committees promoted cohesion in neighborhoods that had not been destroyed. Community and religious celebrations also reinforced collective identity: “After the avalanche (...) people were more attracted to religion” (I_R_M_04).

However, resettlement in condominiums led to conflicts between residents in both cases. In Mocoa, there was no psychosocial support to facilitate adaptation: “It has been difficult to live together (...) we didn’t have any psychological support” (I_LL_M_01). However, some accounts show gradual adjustment processes based on informal norms, social sanctions, and neighborhood cooperation: “We took turns providing security (...) people began to get to know each other” (I_R_M_02).

In Salgar, psychosocial support for adaptation did exist, with the creation of coexistence manuals, administrative boards, and community committees in the resettlement projects. However, these processes were temporary and lost legitimacy after the institutional withdrawal. Persistent conflicts are attributed to the architectural design and the horizontal property regime, which increase daily friction, as well as to the perception of police ineffectiveness in mediating conflicts. As a result, collective initiatives became fragmented and community projects lost continuity once external support ended: “When the foundation was there, they held meetings, but after that, no one cared anymore” (I_R_S_04).

In summary, in Mocoa, despite initial fractures associated with population dispersion and resettlement, binding social capital, supported by schools, churches, and community organizations, together with cultural and religious practices, has favored a partial recomposition of the social fabric. In Salgar, there were psychosocial interventions aimed at promoting coexistence, but their effects were short-lived. The persistence of conflicts, low community participation, and the loss of emotional ties suggest that in post-disaster reconstruction, material infrastructure was prioritized over the relational dimensions of community life.

#### 3.3.3 Recovery of housing

In Salgar, 309 homes had been delivered to affected families within 22 months of the disaster. Both institutions and residents identify the speed of the process as a key factor in resilience, attributed to effective coordination between public and private actors. While the state acted swiftly, private companies fulfilled their contracts. For many families, the new homes represented a substantial improvement over their previous conditions, with larger size, better materials, greater security, and less exposure to risk, which led them to reframe the tragedy as an opportunity to improve their quality of life: “The torrential flood was a very difficult thing. But for me, it turned out to be a blessing...” (FG_R_S_02). However, there were exclusions due to census errors and technical and environmental issues in some projects. In areas such as the La Florida urbanization, leaks and problems with location on unsuitable land were reported: “It was built on top of a lagoon... it had already been warned that it could not be urbanized there” (I_LL_S_02). In addition, for households whose livelihoods are linked to agriculture or animal husbandry, relocation meant a deterioration in their living conditions.

In Mocoa, the situation has been more critical. Of the 1,200 homes planned, only 300 were delivered, leaving 900 families without a permanent housing solution. Although the first homes were delivered 16 months after the disaster, more quickly than in Salgar and under the same national government, during the two subsequent administrations no more have been built, despite the fact that the UNGRD was responsible for the process in all periods. Some beneficiaries expressed satisfaction with the first homes delivered: “We are very happy with the house (...) it has three bedrooms” (I_R_M_02). However, others pointed out limitations in size, the inability to keep animals, and alleged favoritism in the allocation process. For the remaining 900 families, rental subsidies have been insufficient and intermittent; their allocation, maintenance, and increase were the result of citizen protests.

The failure to deliver the homes is attributed to planning errors (purchase of land with environmental restrictions), contracting companies without financial capacity, weak state enforcement of contractual compliance, high turnover of officials, and increased material costs. Social leaders interpret these failures as an expression of patronage practices that privilege private interests over the general interest. In response, the community in Mocoa mobilized its leaders and community organizations to develop citizen oversight processes, public complaints, and protests, managing to halt some irregular contracts and reinstate 400 families that had been excluded from the list of beneficiaries. However, these actions have not been enough to unblock the construction of the missing homes: “People are begging for a rental subsidy (...) that is the pain of not having been compensated” (I_EA_M_01).

In La Primavera, where regulations prohibit public investment in high-risk informal settlements, the reconstruction of homes damaged by river erosion has depended almost exclusively on community support. Affected families receive temporary accommodation from their neighbors, help with cleaning, loans for basic services, and direct assistance with reconstruction, demonstrating the strength of binding social capital.

In summary, while Salgar saw rapid delivery of housing, supported by social capital that connected the population with the state and the private sector, in Mocoa institutional inefficiency, administrative discontinuity, and patronage have blocked the housing solution. The case of La Primavera shows that, in the absence of the state, community social capital can sustain solidarity responses, but it is insufficient in the face of large-scale disasters. In short, these cases show that social capital can facilitate housing solutions when institutional capacity exists and communities can exercise citizen control over projects to curb corruption.

#### 3.3.4 Economic recovery

The torrential downpour severely affected the economies of Salgar and Mocoa, not only due to the loss of homes but also of livelihoods. In both municipalities, the destruction of crops, livestock farms, and family businesses disrupted the local economic base. Social capital, especially family and friendship networks, was key to the immediate economic recovery. Assistance among relatives and friends facilitated the restoration of basic conditions: “There was a stove, a gas pipe (...) he furnished my entire kitchen” (I_LL_M_05).

In La Primavera, where families lack property titles and cannot access state reparations, subsistence relies heavily on mutual support. In this community, the bonds of solidarity and trust to meet the basic needs of the most vulnerable have been constant: “This community is very supportive (...) we are all helping there” (I_LL_P_01). These practices have historical roots in processes of welcoming populations displaced by violence, integrated through networks of care and collaboration: “When we arrived (...) there were mattresses, food (...) everyone wanted to help” (FG_R_P_02). The shared experience of victimization strengthened collective identification and a sense of belonging: “Since everyone was displaced, they were all very supportive of each other” (I_SA_P_02). The case of La Primavera shows that even in conditions of extreme precariousness, families share what little they have, and that solidarity intensifies in the absence of the state. This contrasts with Salgar and Mocoa, where these practices of economic solidarity also exist but appear in a more limited form, given the immediacy of the disaster.

The social capital that connected communities with foundations, companies, and state entities made it possible to channel humanitarian aid in response to the disasters in Salgar and Mocoa. There, state subsidies and compensation were viewed positively: “I bought a washing machine and a refrigerator and started to organize my things” (I_LL_M_05). Loans, training, and support were also provided to entrepreneurs. However, in Mocoa, state support is viewed as a right rather than charity, and as such, it was heavily criticized for its low amount and delays: “It was crumbs (...) a rent subsidy of 200,000 pesos” (I_LL_M_05). In Salgar, meanwhile, there is still a strong dependence on subsidies, reflecting structural vulnerability.

In Mocoa, the lack of permanent housing and insufficient rental subsidies have limited families’ economic progress: “We have been paying for what was lost for six years” (I_EA_M_01). The city’s status as the departmental capital has served as a partial palliative, thanks to the availability of government jobs. However, this status cannot compensate for the fragility of the local productive apparatus: “There are no businesses here (...) except for odd jobs” (I_LL_M_01). In contrast, Salgar continues to depend on the coffee market, which is characterized by instability, forcing residents to adopt informal and flexible subsistence strategies: “One sells empanadas (...) I’m going to sell lunches” (I_R_S_05).

In Mocoa, economic recovery measures, such as bank relief, failed to meet the population’s expectations, and the economic situation was exacerbated by delays in reconstruction work and the COVID-19 pandemic. The reconstruction of Mocoa, initially conceived as an engine for regional development, was carried out in a centralized manner. Resources and contracts were concentrated in the UNGRD and external consortia, frustrating expectations of local economic revitalization: “The resources stayed in Bogotá” (I_LL_M_01). Community leaders denounce patronage, corruption, and the exclusion of local professionals from reconstruction and mitigation works: “95% have been bad contractors” (I_LL_M_14). The local population was marginalized or subcontracted under precarious conditions, with unpaid wages, which led to debt, job insecurity, and institutional mistrust: “There were more than 300 people hired (...) and they weren’t paid” (I_EA_M_01).

In short, in Salgar and Mocoa, binding, bridging, and linking social capital has been fundamental to economic recovery, but it has not been able to compensate for the structural limitations of local productive systems. In Mocoa, reconstruction, which promised economic recovery, was perceived as centralized, inefficient, and riddled with corruption; in Salgar, recovery remains tied to informality and the volatility of the coffee market. Finally, La Primavera shows how binding social capital, historically strengthened by the experience of armed conflict and exclusion, allows recurring crises to be faced with resilience, even if extreme vulnerability persists.

#### 3.3.5 Disaster risk prevention and mitigation: infrastructure works, land use planning, environmental activism, and community practices that reduce disaster risk

In Salgar, risk reduction actions have focused on specific mitigation works (retaining walls, channel management, retention ponds, and expansion of the floodplain) which, although considered necessary, are perceived as insufficient given the magnitude of the threat. The fact that the municipality is unable to invest in new works, adequately maintain existing ones, or manage access to departmental or national funding reinforces a persistent sense of vulnerability. The external support received after the torrential flooding weakened over time, maintaining support for the SAT but not for larger-scale preventive strategies.

In Mocoa, the construction of a large-scale integrated mitigation system has generated considerable controversy. Communities question flaws in planning, execution, and maintenance, as well as the imposition of technocratic designs that did not incorporate local knowledge or the geomorphological conditions of the Amazonian foothills. These tensions reveal weak risk governance, characterized by the absence of effective mediation between local knowledge, experts, and decision-makers. Although citizen participation, an expression of binding social capital, made it possible to partially correct some technical flaws through complaints and social control, this did not translate into legitimacy for the projects, but rather into a critical interpretation that emphasizes their negative social and environmental impacts and their potential to increase risk by operating as unfinished and unmaintained infrastructure. In this context, the works have contributed more to institutional mistrust than to strengthening community resilience.

These contrasts show that mitigation works are not neutral: their ability to contribute to resilience depends on the configuration of social capital. While in Salgar they operate as incomplete but relatively legitimate devices, in Mocoa they become sources of conflict, illustrating how weak risk governance can neutralize, or even reverse, the expected benefits of large-scale technical interventions.

The land use planning processes in Salgar, Mocoa, and La Primavera reinforce this idea. In all three cases, community resilience does not depend solely on the existence of regulatory instruments, but also on their articulation with social dynamics and institutional capacities. Land use planning emerges as a field of dispute between technical approaches, fiscal constraints, and community demands for permanence in the territory or dignified resettlement.

In all three locations, the Watershed Management Plans (POMCA) and Basic Land Use Plans (PBOT) are the public policy instruments that incorporate preventive elements of disaster risk management. Citizens have had some involvement in the formulation of these policies; however, in all three cases, the high proportion of the territory located in threat zones structurally limits their effectiveness: The mass resettlement of families who remain at risk is fiscally unfeasible, and alternative measures are insufficient. Furthermore, in Salgar and Mocoa, there has been some reoccupation of high-risk areas (not by those directly affected by the torrential flood, but by other groups), and in La Primavera, the settlement has continued to grow in an uncontrolled manner. This is due to factors such as the economic vulnerability of the population, migratory flows, and institutional inability to enforce land use policies, among others.

Unlike Mocoa, where some communities in highly cohesive neighborhoods resist preventive resettlement for fear of damaging their social fabric or suffering economic losses, the majority consensus in La Primavera is oriented toward preventive resettlement as a condition for a dignified and safe life. In fact, the community has been supported by cooperation organizations that have provided technical assistance and support to raise awareness of their resettlement project among state authorities, opening up opportunities to incorporate resettlement into larger-scale infrastructure projects. However, to date, the project remains on paper; the economic and political conditions to carry it out have not been met.

In both Salgar and La Primavera, behaviors that increase vulnerability persist, such as uncontrolled burning, but community practices that support risk reduction have also been identified. For example, in La Primavera, community construction of runoff channels, water channeling, stream cleaning, and the transmission of norms to new residents have been identified as practices that are sustained by informal mechanisms of social regulation, self-management, and collective learning. These actions are complemented by a strategic activation of social capital, which has made it possible to influence the removal of dangerous infrastructure in the territory (disused gas pipelines and dangerous electrical infrastructure). In Salgar, there are specific cases in which some residents have effectively incorporated the technical recommendations of the authorities, thereby reducing recurring risks on their properties.

In Mocoa, risk reduction practices are mainly expressed through environmental activism. Bridging social capital manifests itself in networks that integrate technical knowledge, indigenous knowledge, and digital tools, while bonding capital is expressed in grassroots practices focused on environmental care. The core of collective action lies in social capital, particularly in the legal defense of the territory against extractive projects (large-scale mining) perceived as direct threats. However, the conflictive relationship with the state, the asymmetry of power vis-à-vis mining companies, and the criminalization of social leaders have limited the effectiveness of these strategies and generated processes of social polarization that weaken bonding capital in some territories.

The cases show that disaster risk reduction takes different forms depending on the combination of available social capital and institutional response. Strengthening social capital can partially offset infrastructure limitations, while its erosion can increase risk, even in contexts of high economic investment in projects such as mitigation works.

## 4 Discussion

The contributions of the three types of social capital to community resilience in the face of disasters are discussed below.

### 4.1 Contributions from binding social capital

Binding social capital contributed significantly to risk awareness through collective learning based on social memories of disasters and local knowledge about the territory. In the case of Mocoa, the explicit vindication of collective memory was identified as the basis for risk awareness, in line with studies that highlight that memories of past crises can be preserved and transmitted through social capital (4).

Likewise, binding social capital strengthened the SATs. Community members developed daily practices for monitoring water bodies, which complemented institutional mechanisms. As previous research has pointed out, the shared interpretation of threats and vulnerabilities was based on both technical knowledge and everyday experiences and local knowledge (4).

Faced with the failures of the SAT’s technological devices, community networks based on trust, solidarity, and direct communication established themselves as a more sustainable basis for transmitting alerts. In contexts such as La Primavera, these networks not only facilitated the circulation of information in critical situations, but also provided emotional support during evacuation processes. These findings are consistent with the literature indicating that social capital promotes more effective and reliable risk communication, which increases the likelihood that communities will take appropriate measures in response to threats and that, in extreme conditions, family and neighborhood networks tend to become the main source of information, above and beyond official channels (4). Hence, Aguilar (35) describes the relational and communicative processes in their different basic forms, as the support of community resilience.

It was also observed that new residents in communities, especially migrants who were less integrated into community networks, showed lower levels of ownership of risk management plans. This highlights that social cohesion and social memory are key conditions for the sustainability of preparedness processes (4).

During the disaster response in Mocoa and Salgar, close ties between family members, neighbors, and friends, together with local civic institutions (churches, schools, and community organizations), played a central role in providing food, shelter, emotional support, searching for missing persons, and restoring basic services. In La Primavera, where legal restrictions limit public investment, the reconstruction of homes affected by river erosion has depended almost exclusively on community support. These results are consistent with studies that highlight the role of social capital in search and rescue, access to material and financial resources, food provision, shelter, and emotional support (4, 16); all of which demonstrates the organizational capacities of the communities (14, 15) and the collectivization of resources (35), without which it would be impossible to cope with adversity in contexts of high structural vulnerability.

However, support derived from binding social capital tended to concentrate on closer ties, excluding more distant neighbors, confirming that this type of capital can also reinforce dynamics of exclusion (24). Furthermore, these expressions of solidarity were most intense immediately after disasters and weakened in the medium term.

The study showed that neighbourhoods in Mocoa with greater social cohesion and cultural identity, such as those inhabited by indigenous communities, reported more neighborhood support, reinforcing the idea that sociocultural characteristics mediate the relationship between social capital and community resilience (4). Similar findings have been reported in indigenous communities in Fiji, where a strong collective identity is associated with higher levels of social capital and resilience (2).

However, the disasters also eroded social capital in Salgar and Mocoa. The resettlement of families and the breakdown of previous ties weakened networks of trust, generated feelings of loneliness, loss of “neighborhood culture,” decreased community participation, and conflicts in resettlement projects. These results are consistent with research showing the negative effects of disasters on community relations and levels of social capital over time (3, 21). However, the recovery of social capital was facilitated by institutional remnants (schools, churches, and community organizations) and cultural practices, such as traditional and religious celebrations in Mocoa, which reinforced social cohesion, in line with the findings of Aldrich and Meyer (25) and Zhao et al. (4). Therefore, bonding social capital is damaged by the disaster, but what remains of it is, in turn, the basis for rebuilding community relations.

In Mocoa, conflicts between residents in the new settlements were gradually reduced through collaborative work on common problems, such as community surveillance practices, which fostered familiarity and cooperation among neighbors. These findings are consistent with classic social psychology studies that highlight the effectiveness of superordinate goals and intergroup contact in reducing conflict (36). In contrast, in Salgar, the effects of psychosocial interventions did not last after external support was withdrawn, revealing more fragile bonding social capital, constrained by weak leadership, organization, and community participation, and a history of prolonged exposure to armed conflict (37).

The binding social capital that gave rise to emotional support practices among neighbors also played a significant role in mental health recovery in Mocoa and Salgar, coinciding with the systematic review by Hall et al. (3), which identifies its protective function against post-traumatic stress, anxiety, and psychological distress. However, some studies warn that high levels of social capital may also be initially associated with greater stress and anxiety due to the overload of supporting others, a relationship that tends to reverse in later stages of recovery (38, 39). The findings of this study complement the previous explanation by pointing out that in highly cohesive neighborhoods, due to the fact that they are composed of households with kinship ties to each other, multiple bereavements resulting from the density of human losses can intensify long-term psychological suffering for survivors.

In Mocoa, faced with a state response perceived as insufficient, community and volunteer organizations partially compensated for institutional deficits through rescue efforts, parallel censuses, solidarity-based redistribution of aid, and citizen mobilization. These processes reinforced new leadership and expressions of collective action, in line with studies that highlight the role of bonding social capital in community resilience through the strengthening of volunteerism and civic responsibility (4, 24). Strategies that are also replicated in post-disaster scenarios, in response to the distrust in state institutions to address crises quickly, efficiently and promptly (16). A significant contribution of this study is to highlight the contribution of bonding social capital to the risk mitigation phase, an area that has been less explored in the literature, which has traditionally focused on preparedness, response, and recovery (4). In the three communities, self-managed practices of environmental education, land care, and community practices that contribute to risk reduction were identified, such as water channeling and stream cleaning, based on collective learning mechanisms and community participation.

### 4.2 Contributions from bridge capital

Social capital played an important role in post-disaster recovery processes. In the cases of Mocoa and Salgar, this type of capital facilitated access to humanitarian aid and was enhanced by extensive media coverage of the events, which amplified support networks beyond the local sphere. Likewise, bridging social capital contributed to social learning about disaster risk management through knowledge transfer promoted by non-governmental organizations and private actors.

Bridge capital also contributed to risk prevention practices. Activism against large-scale mining, in particular, is sustained by heterogeneous social networks made up of local and external actors who contribute diverse resources, such as technical and legal expertise, indigenous knowledge, and digital tools. This articulation of knowledge constitutes a key basis for social learning that strengthens community resilience in the face of disasters, as has been pointed out in previous studies (4). These processes of collective action involve a plurality of actors, including urban citizens, non-governmental organizations, universities, and indigenous councils, and have made it possible to challenge and limit extractivist interests through the creation and defense of land-use planning instruments. In line with the literature, the findings confirm that bridging social capital facilitates the incorporation of new information, ideas, and knowledge, promoting learning processes, decision-making, and cooperation among socially diverse groups (24).

### 4.3 Contributions from linking social capital

During the disaster response in Mocoa and Salgar, communities benefited greatly from bonding social capital; however, the magnitude of the events overwhelmed local capacities and resources. In this context, bridging social capital played a central role in the emergency response, recovery, and post-disaster reconstruction by providing technical capacities, material resources, and specialized support. However, in contexts such as Salgar, characterized by low levels of community organization and participation, the magnitude of external aid tended to reinforce patterns of dependence on the state.

The three communities made significant progress in building capacity for disaster preparedness and response, mainly through training processes promoted by state entities and cooperation organizations. This strengthening was particularly evident in La Primavera, where capacities were developed in advance, without a prior catastrophic evento. This highlights the role of risk perception, community leadership, and non-welfare-based support from external factors such as NGOs in building anticipatory resilience.

Through bridging social capital, local knowledge was linked with technical knowledge, generating shared frameworks for interpreting risk and transforming beliefs that delegitimize preventive management, such as the idea of disasters as divine punishment. Previous studies have also shown that the integration of local and expert knowledge, mediated by bridging social capital, enhances community resilience to disasters (4).

The social capital network also provided access to key resources to sustain the SATs, obtain specialized advice, access technologies, and maintain two-way communication channels between the state and the community during alert situations. These processes were most effective when external actors offered committed support aimed at strengthening local capacities, rather than welfare-based approaches, which is consistent with previous findings on the role of bridging social capital in accessing information, financial resources, and materials for recovery (40).

However, the contribution of social capital was limited by structural conditions such as low institutional capacity, weak risk governance, and a shortage of economic capital. Despite progress, the three municipalities continue to face constraints in responding to recurring emergencies due to a lack of personnel, resources, and operational capacity of relief agencies, reinforcing perceptions of institutional inefficiency and mistrust toward the State. Humanitarian aid is perceived as palliative and insufficient, which encourages resistance to evacuations and permanence in high-risk areas. In line with other studies, the results show that social capital is a necessary but not sufficient factor for community resilience, which also depends on the availability of economic, physical, human, and natural capital (24). Likewise, the relationship between social capital and community resilience is mediated by variables such as geographical accessibility, livelihoods, income, and the rural or urban nature of the territories (4).

The study identified multiple situations in which low institutional capacity led to limited results in terms of community resilience: delays in rebuilding homes in Mocoa, inability to restrict repopulation of high-risk areas, lack of resources and political will to implement community proposals such as preventive resettlement in La Primavera, and deficiencies in the monitoring of mining activities in Mocoa, among others. These limitations are not solely due to a lack of resources, but also to inter-institutional lack of coordination, centralized decision-making, the disconnect between public policies and social reality (9, 10, 12), corruption, and high turnover among public officials. In this context, although social capital facilitates the allocation of resources for reconstruction and mitigation, it does not guarantee their effective implementation.

Weak risk governance also restricts the potential of bridging social capital by limiting community influence on decision-making. In Mocoa, communities questioned the planning and execution of mitigation works that were perceived as technocratic, poorly contextualized, and lacking in maintenance. These tensions highlight the absence of effective mediation between local knowledge, experts, and policy makers. Although citizen participation made it possible to partially correct some technical flaws in the reconstruction and mitigation projects in Mocoa, it did not translate into institutional legitimacy, but rather into a critical reading of the social and environmental impacts of the interventions. In this regard, other studies have warned that social capital can also limit learning and decision-making when it excludes diverse perspectives and reproduces power asymmetries (24).

Land use planning, which contributes to disaster risk prevention, emerged as a field of dispute between technical approaches, fiscal constraints, and community demands for permanence or dignified resettlement. In Mocoa, collective action relied on social capital to defend the territory legally against extractive projects, particularly large-scale mining. However, the conflictive relationship with the state, the asymmetry of power vis-à-vis mining companies, and the criminalization of social leaders limited the effectiveness of these strategies and, in some cases, weakened binding social capital through processes of polarization.

The results also show that the relative strength of different types of social capital influences resilience outcomes. While bonding social capital predominated in Mocoa, associated with greater progress in rebuilding the social fabric and mental health, bridging social capital was more robust in Salgar, which translated into greater progress in infrastructure and housing and greater satisfaction with these projects. These findings confirm that different forms of social capital have different effects on community resilience to disasters (4).

However, this study contributes to deepening our understanding of the complementary relationships between binding social capital and bridging social capital. In the event of large-scale disasters, when community resources are insufficient, external networks are activated; however, when these supports are deficient or lack transparency, binding social capital, through leadership and community organization, reappears to compensate for institutional deficits and exercise citizen control over reconstruction projects. While post-disaster reconstruction, which often depends on external resources, strengthens local capacities, such as the implementation of SAT, the sustainability of these advances and progress in risk prevention and mitigation depend on continuous efforts that articulate both types of social capital within the framework of collaborative risk governance.

The heterogeneous manifestations of social capital across the three cases reveal critical patterns in how communities mobilize different forms of social connection in response to disaster risk. Table 2 synthesizes the comparative operationalization of bonding, bridging, and linking social capital across Salgar, Mocoa, and La Primavera, illustrating how each type of capital activated differently according to the temporal phase of risk (anticipatory vs. reactive), institutional context, and prior collective trauma. This comparison underscores that social capital is not a uniform resource but rather a dynamic assemblage whose effectiveness depends on the complementarity among its forms and their alignment with governance capacity.

**Table 2:** Comparative manifestation of social capital by case.

Type of social capital	Salgar (Antioquia)	Mocoa (Putumayo)	La primavera (Antioquia -Barbosa)
Bonding social capital	Dense networks of kinship and neighborhood that facilitated immediate solidarity, but amplified emotional pain from multiple losses. Weakened after resettlement due to loss of “neighborhood culture.”	Very strong in neighbourhoods with cultural and indigenous identities, manifested in “mingas” (collective work parties) to restore services and community censuses in response to state failures. Key for social fabric recovery.	High cohesion among families affected by forced displacement. Manifested in constant mutual support for basic needs and artisanal house reconstruction in the absence of state intervention.
Bridging social capital	Spontaneous exchange of resources with unaffected neighborhoods and connections with relatives or companies in other regions to channel humanitarian aid.	Heterogeneous networks integrating local actors with universities and NGOs for environmental activism and legal defense of territory against large-scale mining.	NGO support (Corporación Región) and private companies that helped generate collective awareness of risk and technical studies of the area.
Linking social capital	Highly robust and effective. Fluid coordination between State and private sector enabled rapid housing reconstruction (309 units in 22 months) and high institutional satisfaction.	Weak and conflictive. Marked by distrust due to failure in housing delivery (only 300 of 1,200 units completed), perceived corruption, and centralized reconstruction.	Focused on anticipatory resilience. Seeking state support for preventive resettlement projects, although these often remain “on paper” due to lack of political will or funding.

Source: author’s own elaboration.

## 5 Conclusion

This study reaffirms previous findings and provides new insights into the contribution of social capital to community resilience in the face of disasters, which should be verified in future research.

Overall, the results show that social capital—bonding, bridging, and linking—strengthens community resilience by contributing to risk management in all its phases (prevention/mitigation, preparedness, response, and recovery) through psychosocial processes such as learning and social memory, the articulation between local and technical knowledge, trust, solidarity, community communication, social cohesion, cultural identity, and community empowerment based on leadership, organization, and participation. This contributes to closing the gap in the study of psychosocial conditions, given that previous literature has placed greater emphasis on the structural aspects of social capital (23).

The study expands the literature by highlighting the contribution of social capital to the risk prevention/mitigation phase, which has traditionally been less explored. Bonding social capital, especially at the leadership and community organization levels, favoured self-managed practices of environmental education, land stewardship, and disaster risk reduction, while community participation in risk governance improved the quality of mitigation infrastructure projects. In turn, bridge and linking social capital contributed to prevention through environmental activism and the defense of land-use planning instruments that limit large-scale mining extractives.

Likewise, the findings show that community empowerment, combined with non-welfare-based support from external actors, enables communities with no previous experience of large-scale disasters to strengthen their response preparedness processes to levels comparable to those of communities that have been affected by disasters, contributing to anticipatory community resilience.

The study also confirms that, although disasters damage bonding social capital, the remaining bonds form the basis for the recovery of the social fabric through practices of solidarity and shared social norms. However, limits and ambivalent effects of social capital are identified. Strong bonding social capital prior to the disaster can hinder recovery when the high density of kinship ties increases multiple bereavements and long-term psychological suffering. Furthermore, support derived from binding social capital tends to concentrate on the closest ties and weaken over time, except in communities with high cohesion and social identity which, faced with a lack of external support, depend on mutual support on an ongoing basis, although without overcoming structural conditions of economic vulnerability.

The study also shows that, although bridging social capital is key to attracting external support, it can create harmful dependencies and affect long-term community resilience when support follows a welfare-based approach and bonding social capital in terms of community organization and leadership is weak, a situation that in this study was associated with psychosocial trauma resulting from previous experiences of intense socio-political violence. In this sense, communities tend to replicate the collective coping strategies they developed in response to past adversities. One contribution of this study is to demonstrate that when war and psychosocial trauma weaken leadership and organizations, these capacities are not mobilized for risk management; conversely, when the experience of collectively coping with the effects of war strengthened community empowerment, these structures were subsequently activated to address disaster risk.

Finally, the results confirm that social capital is a necessary but not sufficient factor for community resilience, which also depends on other forms of capital and on institutional and governance conditions. In this study, the contribution of bridging social capital was limited by weaknesses in state institutional capacity and risk governance, such as inter-institutional lack of coordination, centralization, low levels of citizen participation, corruption, and high turnover of officials. Therefore, although bridging social capital facilitates the allocation of resources to disaster risk management processes, it does not guarantee their effective implementation.

The differences between the cases analysed show that the relative strength of different types of social capital influences resilience outcomes. In Mocoa, bonding social capital predominated, associated with greater perceived progress in rebuilding the social fabric and mental health; in Salgar, bridging social capital was more robust, favouring progress in infrastructure, housing, and satisfaction with reconstruction projects. These findings confirm the differentiated and complementary effects of different forms of social capital and underscore that the sustainability of community resilience depends on the articulation between bonding and bridging social capital within the framework of collaborative risk governance.

## 6 Limitations

The main limitation of the study is that the data were generated between 2021 and 2023, several years after the torrential floods occurred. Consequently, the information relating to experiences before and during the disaster comes from accounts based on memories and reinterpretations after the event, processes that may be subject to distortions inherent in memory. Therefore, memory bias may affect the reconstruction of the role of social capital by reinterpreting the contribution of certain actors, relationships, or forms of support in light of subsequent experiences. To enhance the validity of the findings, we employed several triangulation strategies. First, we included a diverse range of participants (e.g., community leaders, residents, state actors, NGOs) to contrast perspectives across different social positions. Second, we combined multiple data generation strategies—individual and group techniques, as well as participant observation—together with documentary analysis (e.g., institutional documents and press sources), thereby enabling cross-verification of key events and processes.

As with any qualitative case study, the objective is analytical generalization, not statistical generalization; therefore, the findings are contextual and cannot be directly extrapolated to other communities or types of disasters. Furthermore, the evidence presented is based exclusively on qualitative data; therefore, the results of this study do not account for causal relationships but rather interpretations that must be tested in further future research.

## Data Availability

The raw data supporting the conclusions of this article will be made available by the authors, without undue reservation.

## Footnotes

1 To ensure the anonymity of the 96 interviewees and participants in the 10 focus groups, a coding system is employed that identifies the data collection instrument (I=Interview/FG = Focus Group), the type of actor (LL = Local Leader/R = Resident/SA = State Actor/EA = External Actor outside state and society, such as private companies and non-governmental organizations (NGOs)/ER = Emergency Response Personnel), and the case study (S=Salgar/M = Mocoa/P = La Primavera).

## References

[1] Chen C Xu L Zhao D Xu T Lei P. A new model for describing the urban resilience considering adaptability, resistance and recovery. Saf Sci. (2020) 128:104756. doi: 10.1016/j.ssci.2020.104756

[2] Nakamura N Kanemasu Y. Traditional knowledge, social capital, and community response to a disaster: resilience of remote communities in Fiji after a severe climatic event. Reg Environ Chang. (2020) 20:23. doi: 10.1007/s10113-020-01613-w

[3] Hall CE Wehling H Stansfield J. Examining the role of community resilience and social capital on mental health in public health emergency and disaster response: a scoping review. BMC Public Health. (2023) 23:2482. doi: 10.1186/s12889-023-17242-x, 38082247 PMC10714503

[4] Zhao G Hui X Zhao F Feng L Lu Y Zhang Y. How does social capital facilitate community disaster resilience? A systematic review. Front Environ Sci. (2025) 12:1496813. doi: 10.3389/fenvs.2024.1496813

[5] Ziglio E. Strengthening Resilience: A Priority Shared by Health 2020 and the Sustainable Development Goal. Geneva: World Health Organization.

[6] Rojas-Betancur MA Posada-Pérez N Londoño Torres GE. Vulnerabilidad Institucional y Resiliencia Comunitaria en la Gestión del Riesgo de Desastres: Estudio de Caso Comparado en Antioquia, Colombia. REDER. (2026) 10:40. doi: 10.55467/reder.v10i1.206

[7] Cuthbertson J Archer F Robertson A Rodriguez-Llanes J. A socio-health approach to improve local disaster resilience and contain secondary crises: a case study in an agricultural community exposed to bushfires in Australia. Prehosp Disaster Med. (2023) 38:3–10. doi: 10.1017/S1049023X22002436, 36606323 PMC9885428

[8] Moreno J Lara A Torres M. Community resilience in response to the 2010 tsunami in Chile: the survival of a small-scale fishing community. Int J Disaster Risk Reduct. (2019) 33:376–84. doi: 10.1016/j.ijdrr.2018.10.024

[9] Morán RCD Dávila LV Gómez HEL. Política para acentuar la resiliencia social: gestión del riesgo ante desastres. Revista de Filosofia (Venezuela). (2022) 39:674–87. doi: 10.5281/zenodo.7067501

[10] Aldunce P Beilin R Howden M Handmer J. Resilience for disaster risk management in a changing climate: practitioners’ frames and practices. Glob Environ Change. (2015) 30:1–11. doi: 10.1016/j.gloenvcha.2014.10.010

[11] Cárdenas M Bonilla JP Brusa F. In: Funaro R, editor. Climate Policies in Latin America and the Caribbean: Success Stories and Challenges in the Fight against Climate Change. Washington: Inter-American Development Bank (2021)

[12] Insulza J Jiménez A Cerda C. Resiliencia urbana multidimensional en contextos de riesgo: estrategias para el Programa “Quiero Mi Barrio” desde el caso “Barrio Olga Leiva” en Peñalolén. EURE. (2023) 50:10. doi: 10.7764/EURE.50.149.10

[13] Otálora Barreto ZI Sánchez Barreto RF. La organización social comunitaria como estrategia de reducción de la vulnerabilidad humana ante el colapso planetario. CPL. (2025) 10:1–27. doi: 10.35600/25008870.2025.21.0357.1

[14] Delilah Roque A Pijawka D Wutich A. The role of social capital in resiliency: disaster recovery in Puerto Rico. Risk Hazard Crisis Pub Pol. (2020) 11:204–35. doi: 10.1002/rhc3.12187

[15] Muñoz-Duque LA Navarro O Restrepo-Ochoa D Fleury-Bahi G. Risk perception and trust management in inhabitants exposed to coastal flooding: the case of Cartagena, Colombia. Int J Disaster Risk Reduct. (2021) 60:102261. doi: 10.1016/j.ijdrr.2021.102261

[16] Rodríguez Otálora JA Garzón Santos JL. Perspectivas tecnológicas para la gestión del riesgo en el sector solidario en Colombia. criteriolibre. (2025) 22:31–47. doi: 10.18041/1900-0642/criteriolibre.2024v22n41.11950

[17] Coleman JS. Social capital in the creation of human capital. Am J Sociol. (1988) 94:S95–S120.

[18] Putnam RD. Bowling alone: America’s declining social capital. J Democracy. (1995) 6:65–78. doi: 10.1353/jod.1995.0002

[19] Woolcock M. The place of social capital in understanding social and economic outcomes. Can J Policy Res. (2001) 2:11–7.

[20] Caldwell K Boyd CP. Coping and resilience in farming families affected by drought. Rural Remote Health. (2009) 9:1088 19415964

[21] Wong H Huang Y Fu Y Zhang Y. Impacts of structural social capital and cognitive social capital on the psychological status of survivors of the Yaan earthquake. Appl Res Qual Life. (2019) 14:1411–33. doi: 10.1007/s11482-018-9661-9

[22] Susanto IW Kusumasari B Santoso AD Bafadhal OM. Social capital in disaster management: a systematic literature review of research trends from 1998 to 2019. Indonesian J. Geography. (2023) 55:179. doi: 10.22146/ijg.71572

[23] Carmen E Fazey I Ross H Bedinger M Smith FM Prager K . Building community resilience in a context of climate change: the role of social capital. Ambio. (2022) 51:1371–87. doi: 10.1007/s13280-021-01678-9, 35015248 PMC9005590

[24] Aldrich DP Meyer MA. Social capital and community resilience. Am Behav Sci. (2015) 59:254–69. doi: 10.1177/0002764214550299

[25] Slife BD Christensen TR. Hermeneutic realism: toward a truly meaningful psychology. Rev Gen Psychol. (2013) 17:230–6. doi: 10.1037/a0032940

[26] Yin RK. Case Study Research and Applications: Design and Methods. Los Angeles: SAGE (2018).

[27] Stake RE. Qualitative case studies. En: Denzin NK Lincoln YS, editors. The Sage Handbook of Qualitative Research. London: Sage Publications Ltd; (2005).

[28] Mulia FA Handayani W. Assessment and comparison of community resilience to floods and tsunamis in Padang, Indonesia. IDRiM J. (2024) 14:115826. doi: 10.5595/001c.115826

[29] OECD. Evaluación de la Gobernanza del Riesgo en Colombia. Paris: OECD Publishing (2019).

[30] Merriam SB. Qualitative Research and Case Study Applications in Education. San Francisco: Jossey-Bass Publishers (1998).

[31] Saldaña J. The Coding Manual for Qualitative Researchers. Thousand Oaks: SAGE Publishing Inc (2021).

[32] Sánchez Steiner LM. La Ciudad-Refugio: Migración Forzada y Reconfiguración Territorial Urbana en Colombia: El Caso de Mocoa. Barranquilla: Universidad del Norte, Editorial; Consejo Profesional Nacional de Arquitectura y sus Profesiones Auxiliares (2012).

[33] Centro de Fe y Culturas, Conciudadanía. Suroeste Antioqueño: Un Conflicto Silenciado. Aproximación a la Construcción de Memoria Histórica del Conflicto Armado en el Suroeste Antioqueño (1984–2016). Medellín: Centro de Fe y Culturas y Conciudadanía (2020).

[34] Aguilar Llanto M. Procesos de comunicación organizacional y resiliencia comunitaria en contextos de desastres socionaturales en América Latina: una revisión sistemática de literatura. SL. (2025) 9:e2517. doi: 10.26490/uncp.sl.2025.9.1.2517

[35] Girón López LA. Resiliencia comunitaria, cuidado comunitario y pandemia en una olla común de Lima. RevPsicología. (2025) 34:80281. doi: 10.5354/0719-0581.2025.80281

[36] Ovejero A. Estereotipos, prejuicios y discriminación. En: Psicología Social, Algunas Claves Para Entender el Comportamiento Humano. Mexico City: Biblioteca Nueva; (2010).

[37] Londoño PAV Monsalve LFD Trujillo SP. The significance of prior resilience experiences in communities exposed to socio-political violence in Colombia for enhancing the community-based disaster management (CBDM) model. J Infrastructure Policy Devel. (2024) 8:5352. doi: 10.24294/jipd.v8i9.5352

[38] Weil F Lee MR Shihadeh ES. The burdens of social capital: how socially-involved people dealt with stress after hurricane Katrina. Soc Sci Res. (2012) 41:110–9. doi: 10.1016/j.ssresearch.2011.06.006, 23017700

[39] Lowe SR Sampson L Gruebner O Galea S. Psychological resilience after hurricane Sandy. PLoS One. (2015) 10:e0125761. doi: 10.1371/journal.pone.0125761 25962178 PMC4427458

[40] Birhanu Z Ambelu A Berhanu N Tesfaye A Woldemichael K. Understanding resilience dimensions and adaptive strategies to the impact of recurrent droughts in Borana zone, Oromia region, Ethiopia: a grounded theory approach. Int J Environ Res Public Health. (2017) 14:118. doi: 10.3390/ijerph14020118, 28134771 PMC5334672

