# Supplementary material for: Social capital and community resilience in disaster contexts: a multiple case study from Colombia
Source: Front Public Health. 2026 May 26;14:1830318. doi: 10.3389/fpubh.2026.1830318 (PMC13246728; doi:10.3389/fpubh.2026.1830318)
Supplement: Supplementary file 1 [file Table_1.DOCX]

**Supplementary Material 1**

**Category Map**

**DISASTER**

- Event memory
  - Torrential flash flood
  - Flooding
- Impacts
  - Human
  - Health
  - Community relations
  - Material

VULNERABILITY

- Social
  - Socio-political violence
  - Forced displacement
  - Crime
  - Migration
  - Informal settlement
  - COVID-19 pandemic
  - Psychosocial trauma
  - Mining exploitation
  - Accelerated urbanization
  - Land use
- Physical
  - Geological
  - Hazardous infrastructure
- Political
  - Systemic corruption
  - Clientelism
  - Political opportunism
- Economic
  - Low income levels
  - Unemployment
  - Low industrialization
  - Inflation
  - Inadequate housing

**RISK MANAGEMENT**

- Risk knowledge
  - Foretold tragedy
  - Technical risk assessment
  - Social perception of risk
  - Risk communication
- Emergency preparedness and response
  - Disaster risk management (DRM) training
  - Drills
  - Community DRM plans
  - Evacuation routes
  - Equipment/materials
- Prevention
  - Mitigation works
  - Land-use planning
  - Preventive resettlement
- Emergency response
  - Relief
  - Social support
  - Psychosocial support
  - Restoration of services
  - Humanitarian aid
- Post-disaster recovery
  - Physical and mental health
  - Social fabric
  - Infrastructure
  - Housing
  - Livelihoods

COMMUNITY

- Psychosocial processes
  - Cultural identity
  - Community communication
  - Sense of community
  - Social cohesion
  - Collective memory
  - Solidarity
  - Community organization
  - Social leadership
  - Trust
  - Collective learning
- Social capital
  - Bonding
  - Bridging
  - Linking

STATE INSTITUTIONALITY

- Capacity
  - Financial resources
  - Infrastructure
  - Knowledge
  - Public policies
- Political will
- Internal coordination
- Control and oversight
- Administration of justice

POLITICAL SUBJECTIVITIES

- Political motivations
  - Improving quality of life
  - Vocation for service
  - Pursuit of equity/justice
  - Altruism
  - Civic commitment
  - Territorial identity
  - Belief in collective action
  - Personal growth
  - Social recognition
  - Sense of agency
- Political socialization
  - Family tradition
  - Mentorship
  - Civic/political education
  - Politicization of adversity
  - Leadership trajectory
- Political discourses
  - Environmentalism
  - Social justice
  - Development models
- Empowerment
  - Visibility in the public sphere
  - Claiming rights
- Political emotions
  - Public love
  - Indignation

CITIZEN PARTICIPATION

- Modes
  - Conventional
  - Non-conventional
  - Community-based
  - Volunteering
  - Environmental activism
- Resources
  - Civic skills
  - Knowledge
  - Time
  - Economic autonomy
  - Funding for community projects
- Internal weaknesses
  - Intra-community conflicts
  - Disputes among leaders
  - Gender inequalities
- Participation fatigue
  - Low community engagement
  - Frustration
  - Tensions between private and public life
  - Lack of recognition

RISK GOVERNANCE

- Institutional barriers to citizen participation
  - Invisibility
  - Exclusion from decision-making
  - Consultation without deliberation
  - Disregard for local knowledge
  - Manipulation
  - Inaccessible public information
  - Centralization
  - Intimidation of leaders
  - Usurpation
  - Co-optation
- Impact of citizen participation
  - Not real
  - Symbolic
  - Citizen power
- Types
  - Top-down
  - Bottom-up
  - Collaborative

COMMUNITY RESILIENCE

- Meanings
- Social perception
- Types
  - Reactive
  - Anticipatory
  - Transformative
- Citizen contributions
  - Self-managed resilient infrastructure
  - Everyday risk-reducing practices
  - Improved quality of DRM projects
  - Increased transparency in DRM projects
  - Sustainability of DRM processes
  - Reconstruction of the social fabric
  - Reduction of vulnerability
- Citizen agency
  - Self-management
  - Cooperation
  - Compensation
  - Demand-making
  - Social oversight
- Other domains
  - Family
  - Individual
